# Three-year trajectories in functional limitations and cognitive decline among Dutch 75+ year olds, using nine-month intervals

**DOI:** 10.1186/s12877-021-02720-x

**Published:** 2022-02-01

**Authors:** Maura Kyra Maria Gardeniers, Marjolein Irene Broese van Groenou, Erik Jan Meijboom, Martijn Huisman

**Affiliations:** 1 Department of Sociology, Vrije Universiteit Amsterdam, De Boelelaan, 1081 Amsterdam, The Netherlands; 2 Department of Epidemiology & Biostatistics, Amsterdam Public Health research institute, Amsterdam UMC, Vrije Universiteit Amsterdam, De Boelelaan, 1117 Amsterdam, Netherlands

**Keywords:** Trajectories, Functional limitations, Cognitive decline, Mortality, Group-based trajectory modelling

## Abstract

**Background:**

Using longitudinal panel data, we aimed to identify three-year trajectories in cognitive and physical functioning among Dutch older adults, and the characteristics associated with these trajectories.

**Methods:**

We used Group-based Trajectory Modelling with mortality jointly estimated to identify trajectories, using a scale composed of 6 Activities of Daily Living (ADL) as a measure of physical functioning, and the short mini mental status examination (sMMSE) or the Informant Questionnaire on Cognitive Decline in the Elderly (IQCODE) as a measure of cognitive functioning. Data came from 574 Dutch adults aged 75+, collected in five nine-month measurement waves (2015–2018) for the Longitudinal Aging Study Amsterdam.

**Results:**

For physical functioning five trajectories were identified: ‘high’, ‘moderate’, ‘steeply declining’, ‘gradually declining’, and ‘continuously low’; and for cognitive functioning: ‘high’, ‘moderate’, ‘declining’, and ‘low’. Living in an institution, and being lower educated increased the probability of the two continuously low functioning trajectories, whereas old age and multimorbidity increased the probability of low physical functioning, but multimorbidity decreased the probability of low cognitive functioning. Associations for steeply declining physical functioning were absent. Being older and having multimorbidity increased the probability of gradually declining physical functioning and declining cognitive functioning. A higher prevalence of lung- and heart disease, cancer, and rheumatic disease was found in the gradually declining physical functioning group; and a higher prevalence of diabetes, cerebrovascular accidents, and cancer was found in the declining cognitive functioning group. High and moderate physical functioning and high cognitive functioning were characterized by being younger, community-dwelling, and higher educated. Having multimorbidity negatively predicted high and moderate physical functioning, but was not associated with high and moderate cognitive functioning.

**Conclusions:**

This study identified trajectories comparable to studies that used longer time intervals, showing the consistent presence of heterogeneity in both physical and cognitive trajectories. Co-modelling mortality resulted in bigger group sizes for the more adverse trajectories. The favourable trajectories, containing most of the participants, were mostly characterized by absence of disease. The prevalence of chronic diseases differed between the declining trajectories, suggesting that certain diseases tend to induce cognitive decline rather than physical decline, and vice versa.

**Supplementary Information:**

The online version contains supplementary material available at 10.1186/s12877-021-02720-x.

## Background

Western societies are ageing rapidly, showing both an increase in the number of young older adults (aged 65 to 75) and the number of oldest-old (aged 75 and older) [1]. For some older adults this increased longevity goes hand in hand with chronic disease, functional limitations, and cognitive decline [2–6]. But ageing does not appear to be debilitating to all, and some older adults reach high age while maintaining good physical and cognitive functioning [7]. In other words, there is considerable heterogeneity in health and functioning in old age. Exploring this heterogeneity is a fundamental task of gerontological research. Since levels of functioning are known to be dynamic and not static, information about trajectories of functioning is key for understanding how functioning is associated with ageing. Depending on various aspects of study design- such as the chosen indicator of functioning, the density of observation points, and the age of the population studied- previous studies identified between two and nine trajectories of physical or cognitive functioning [7–20]. Despite this wide variety in the number of trajectories observed, the trajectories consistently differed based on age. So, the studies that focussed on older populations tend to be consistent in that they often report trajectories with high levels of functioning, mostly report trajectories with decline, but do not report trajectories reflecting recovery of functioning [16, 21, 22]. Studies focussing on younger populations (~ 55–75 years) sometimes report recovery or fluctuating trajectories [13, 23–25], whereas studies that focus on older populations (75+) tend to report no recovery [16, 21, 22].

Because these previous trajectory studies vary in key aspects of study design, the best approach to gain a more comprehensive understanding of the heterogeneity in trajectories of functioning in older adults may be to combine several design aspects of these studies. Existing studies of functional limitations in Dutch older adults focused on relatively young samples (~mean age 70) and used relatively long time intervals of 3 years to assess trajectories of limitations [7, 12, 13, 21]. In the current study, we investigate heterogeneity in functional limitations and cognitive functioning among Dutch older adults. Our approach stands out from previous work on a number of aspects of study design. Firstly, we examine changes in functioning across relatively short time intervals of nine months, which may be more sensitive to meaningful change than longer follow-up periods. Secondly, we focus on a sample of participants aged 75 years and older, which is where we expect relatively many changes in functioning to occur. Thirdly, we measure functional limitations as a continuous variable. This allows for more accurate descriptions than dichotomization, which severely compresses the range of the severity of functional limitations. Finally, we incorporate information on mortality risk in our estimates of trajectories to account for bias in estimated group sizes caused by nonselective attrition caused by mortality [26]. This approach builds on the assumption that attrition due to mortality is related to the participant’s previous health status and provides relevant information for classifying individuals into broader functioning categories, leading to more accurate classifications. Studies that did not account for mortality in their models implicitly made the assumption that decease occurred at random [26]. In addition, we explore differences in several key characteristics of older adults between each of the observed trajectories. Our research question is as follows: What trajectories in functional limitations and cognitive functioning can be identified in Dutch adults aged 75 and older in a period of three years? And how are age, sex, socioeconomic status, and chronic diseases associated with these trajectories?

## Methods

### Design and study sample

This study used data from the LASA 75-PLUS-study, an ancillary study of the Longitudinal Aging Study Amsterdam (LASA). LASA is an ongoing longitudinal population-based study of older adults (aged 55+) in the Netherlands [27]. The baseline sample was drawn from eleven municipal registries in 1992, stratified by age and sex, and contained 3107 men and women aged 55–84 years (born between 1908 and 1937). In 2002 and 2012 additional cohorts were sampled of respectively 1002 and 1032 men and women born between either 1938 and 1947, or 1948 and 1957. The baseline cooperation rate was 62% for the first cohort, and 62 and 63% for the second and third cohort [27, 28]. The data were mainly collected by trained interviewers in face-to-face, computer-assisted interviews. In cases where respondents refused or were not able to complete the full interview, either an abbreviated face-to-face interview, or a 15-min telephone interview (with either a proxy or the respondent) was conducted. Further details concerning data collection are described in cohort profile papers [27, 28]. Although previous studies excluded demented and institutionalized people [7, 8], we did not exclude participants based on these criteria. We included participants whose scores were low on the cognitive tests, which might indicate the presence of dementia. However, we did not check the presence of a formal diagnosis of dementia. Participants with low cognitive scores were included in the sample because we also aimed to study whether the trajectories in functional limitations and cognitive decline showed overlap. For comparability reasons we wanted the samples in both the analyses to be as comparable as possible. Participants that were censored due to other reasons than death (*N* = 34) were excluded from the analysis because of two reasons. First, this was a very small number compared to the participants that dropped out due to decease (*N* = 139). And second, a similar study conducted by Zimmer et al. [29] showed that the trajectories estimated for the sample including participants that were censored due to other reasons than death looked similar to the trajectories estimated when dropout was solely modelled for deceased participants. This suggests that this data is usually probably missing not at random as well. It seems quite likely that participants refuse an interview due to health issues, but it is also possible that participants missed an interview because of reasons related to exquisite health (such as going on vacation). Because of this uncertainty, and because the number of participants that had missing data for other reasons than death was quite low (*N* = 34), we excluded these people from the analysis. Results might have been less generalisable if we also modelled these people, where the missingness-mechanism would have been rather different, together with people for whom the missingness-mechanism was due to decease.

For the ancillary 75-PLUS-study, three additional nine-monthly measurement waves were conducted between the regular measurement waves of 2015/16 and 2018/19. All LASA-participants born before 1941 were asked to participate in the ancillary study (*N* = 686), of whom 601 eventually participated in the LASA 75-PLUS study. For this study we used these three nine-monthly measurements: 75-PLUSI, 75-PLUSII (*N* = 550) and 75-PLUSIII (*N* = 507), together with data from the preceding (2015/16) and subsequent (2018/19) regular LASA waves (*N* = 473). Table 1 shows the number of participants included in each wave.

**Table 1:** Description of the 5 LASA measurement waves conducted between 2015 and 2019

	Wave 2015–16	Wave 75PLUS I	Wave 75PLUS II	Wave 75PLUS III	Wave 2018–19
Invited	686	686	601	550	473
Participated, n (%)	601	601 (87.6)	550 (91.5)	507 (92.2)	473
Age, mean (SD)	82.2 (5.4)	83.0 (5.4)	83.4 (5.2)	83.8 (4.9)	84.1 (4.8)
Female, n %	368 (61.2)	368 (61.2)	338 (61.5)	311 (61.3)	295 (62.4)
Functional limitations, mean (SD)	23.9 (6.3)	22.7 (6.9)	23.1 (6.3)	22.3 (6.4)	22.4 (6.5)
Cognitive functioning, mean (SD)	14.3 (1.9)	14.6 (1.7)	14.6 (1.8)	14.6 (1.8)	14.2 (2.1)
Face-to-face interview, n	453 (78.0)	442 (73.5)	410 (68.2)	364 (66.2)	326 (68.9)
Telephone interview proxy	53 (8.8)	61 (10.1)	55 (10)	59 (11.6)	50 (10.3)
Telephone interview respondent	86 (14.3)	98 (16.3)	85 (15.5)	84 (16.6)	93 (17.0)

### Dependent variables

#### Functional limitations

Functional limitations are restrictions in performing physical or mental tasks, that usually result in limitations in the performance of activities of daily living (ADL). We used ADL-indicators of respondents’ ability to perform the following six tasks: 1) dressing or undressing themselves, 2) standing up from or sitting down in a chair, 3) cutting own toenails, 4) using own or public transport, 5) climbing a flight of stairs, and 6) walking 5 min outdoors without resting. The response categories ranged from ‘1’ not able at all, to ‘5’ very able. The responses to the ADL-items were summed to the ‘functional-limitations-scale’, that ranged from 6 to 30, with higher scores indicating higher levels of functioning.

#### Cognitive decline

The degree of cognitive decline was assessed using either the sMMSE, a short 8-item version of the Mini-Mental State Examination (MMSE) [30, 31], in which functioning in the following domains was tested: orientation in time and place, registration of words, attention and calculation (measured by either subtraction or spelling), and recall of three words. For participants that were unable to perform the test, cognition was assessed by interviewing a proxy, if possible. For these interviews an abbreviated form of the IQCODE [32] was used: a 6-item scale ranging from 18 to 30 concerning the decline in the last 10 years on remembering 1) conversations, 2) addresses, and 3) phone numbers, and handling 4) domestic appliances, 5) money for groceries, and 6) finances. Higher scores indicated worse decline. For those participants who switched to the IQCODE at some point during the study, we imputed sMMSE data based on the IQCODE-scores. No guidelines have been published on how to harmonize the IQCODE with the sMMSE. Therefore we tried various ways of harmonizing the two, based on two studies that used both scales and reported which scores indicated similar levels of cognition [33, 34]. Since these values differed between these two studies, we estimated the trajectories using these two ways of harmonizing the two scales, to assess whether this affected the shapes of the estimated trajectories. This did not affect the shapes substantially (see Fig. S1 in Additional file 1). Because different cut-off points did not rigorously affect the trajectory shapes, we chose the cut-off points based on a previous LASA-study [34], since this would likely reflect our sample best. The values of the IQCODE and sMMSE are reported in Table 2. Because an IQCODE-score of 18 indicated no change in the last years, this value either corresponded with the participants’ previous sMMSE-score, or if that score was not available, an sMMSE-score of 16. The eventual ‘cognition-scale’ was constructed by summing all the points scored on the 8 sMMSE items (or by harmonizing the IQCODE to an sMMSE-score), resulting in a scale that ranged from 0 to 16, with 16 indicating the highest level of cognitive functioning.

**Table 2:** Values used to harmonize IQCODE-scores with sMMSE-scores, based on cut-off values provided by van den Kommer et al., 2018 [34]

IQCODE	sMMSE
18	16, or previous sMMSE-score
19	16
20–21	15
22–23	14
24–25	13
26–27	12
28–29	11
30	10

Despite there being indications of the IQCODE and MMSE not entirely measuring the same construct [35], we argue that keeping the participants that were assigned the IQCODE at some point during the study in the analyses is better than excluding them. Since our study focusses on cognitive decline, and the IQCODE is more likely to be assessed when participants experience considerable decline, excluding these participants would have likely resulted in missing a considerable portion of the trajectories showing cognitive decline. Since the model aims to be descriptive and not explanatory, we decided that the occurrence of decline was more informative than the rate of decline. Even if the imputed data based on IQCODE-scores is an under- or overestimation of the “true” sMMSE-score, we expect the direction of cognitive decline to still be in accordance with the “true” direction.

#### Mortality

Mortality-data (date of decease) were obtained through the registration of municipalities (GBA), and were last updated in February 2020. It was included as a dichotomous variable, with ‘0’ indicating being alive and ‘1’ indicating being deceased, per wave.

### Independent variables

Age, sex, partner status (partner/ no partner), educational level, and chronic disease status (number of diseases) were used to give a description of respondents in the identified trajectories. This selection of characteristics was chosen because they represent some of the main vectors of social and economic disadvantage in older populations and reflect vulnerable groups. They were all measured at baseline (2015/16). Educational level was divided into three categories: low (primary school), middle (secondary school or lower vocational training), and high (higher vocational training or higher). For chronic disease we grouped ten diseases into five categories: 1) heart- and lung disease (coronary-, pulmonary-, and vascular disease), 2) rheumatic disease (arthritic and osteopathic), 3) diabetes, 4) cancer, and 5) cerebrovascular accidents (CVA), we used these five diseases to construct a variable that measured the number of chronic diseases.

### Sensitivity and missing data

Data was either missing at the item level (e.g. one of the six ADL-items missed), or at the wave level (e.g. not participating in one wave). When a respondent had less than ~ 50% missing at the item level, data were imputed with the respondent’s mean of the non-missing items at that wave. When more than 50% of the items of the outcome was missing, the outcome variable at that wave was imputed with the mean of the preceding and subsequent wave. Data that was missing at the wave level was only imputed when data for the previous and subsequent wave was available. This linear interpolation imputation method used for imputation of the waves tends to provide a good fit for longitudinal missing data [36], and it has been suggested this is even the case if the data are missing not at random [37]. For a more in depth review on the rationale for the mean-imputation method see: Halpin [38]. Data were imputed for 73 participants. Of the initial sample of 601 4.7% (*N* = 34) dropped out for other reasons than decease, and were thus excluded from the analysis. We conducted sensitivity checks, by estimating the models 1) stratified by sex, 2) only for survivors, and 3) for deceased (see Figs. S2, S3, and S4 in Additional file 2).

The models stratified for sex did not differ substantially in terms of shapes of the trajectories, so we performed the main analyses unstratified. The models only for survivors and deceased participants were comparably different, as expected, yet showing the importance of including the deceased participants in the analysis by jointly modelling mortality.

### Method of analysis

#### Group-based trajectory modelling

The analyses were conducted using the STATA package Proc Traj [39]. As suggested by Nagin [40] we started with estimating an unconditional model, in which even chronological age was not included. This approach has the advantage of not allowing one covariate to have a disproportionately big influence on the model. The independent variable in the model was the time of the measurement waves. We fitted two group-based trajectory models with mortality jointly estimated, for physical and cognitive functioning. Because the dependent variables were continuous scales, we used the Tobit model, assuming a censored normal distribution [41]. First, we determined the number of identified trajectories that fitted the data best, by using the Bayesian Information Criterion (BIC) and the posterior probabilities, shown in Table S1 and S2 in Additional file 3 [39, 42]. We also assessed whether an extra trajectory group revealed a relevantly different trajectory. After having identified the optimal number of trajectories, cognitive or physical limitations were estimated in a trajectory model, with the dropout-function accounting for dropout due to decease [26, 43]. This dropout-function is explained in more detail in Additional file 4. The 27 participants who had missing data due to other reasons than death and could not have their data imputed, were excluded from the analysis, using the obsmar-function [44]. Subsequently we calculated the average marginal effects (AME). The AME are a variation on a multinomial logistic regression, and show the association between a certain characteristic (i.e. age) and a trajectory for a one-unit change of that characteristic. The AME’s main advantage over the estimates of multinomial logistic regression lies in the fact that their provided estimates are more intuitive in terms of interpretation since they don’t require a reference group during interpretation [45].

## Results

### Descriptive statistics

The baseline (wave 2015/16) characteristics of the 567 participants are shown in Table 3. The average age was 82.20 years, with 61% being female, 50% currently having a partner, and respectively 48, 30, and 22% having had low, middle, and high education. Over the three years of follow-up 24% (*N* = 139) deceased. For each wave, data was collected by proxy for 12.3 to 15.3% of the sample. Most participants suffered from rheumatic diseases (63%) and heart- and lung disease (49%), whereas less people had diabetes (16%), CVA (10%), or cancer (23%). The average number of diseases was 1.71, 53% had two or more chronic diseases.

**Table 3:** Descriptive statistics per trajectory type of 567 Dutch adults aged 75+

		Functional Limitations					Cognitive functioning			
	***Total sample*** *(N = 567)*	***1:*** *Continuous low functioning. High mortality* *(N = 127)*	***2:*** *Steep decline in functioning. Low mortality* *(N = 36)*	***3:*** *Gradual decline.* *low mortality* *(N = 149)*	***4:*** *Moderately high functioning. Low mortality* *(N = 189)*	***5:*** *Continuous high functioning. Low mortality (N = 66)*	***1:*** *Continuous low functioning. High mortality (N = 60)*	***2:*** *Declining functioning. Moderate mortality (N = 113)*	***3:*** *Moderately high functioning. Low mortality* *(N = 222)*	***4:*** *Continuous high functioning. Low mortality (N = 172)*
%	100	22.0	6.7	26.4	33.3	11.5	10.9	19.5	39.0	30.6
Mean age (sd)	82.20 (5.32)	85.43 (5.80)	82.89 (5.36)	83.25 (5.14)	80.42 (4.15)	78.28 (2.71)	84.92 (5.08)	84.01 (6.04)	81.60 (5.13)	80.82 (4.18)
Age range	74.86–102.86	75.09–102.86	75.20–94.31	75.07–97.59	75.05–98.23	74.86–85.88	75.26–95.73	75.07–102.86	75.00–98.23	74.86–93.25
Comorbidity	1.71 (1.19)	1.97 (1.31)	1.44 (1.11)	2.05 (1.25)	1.53 (0.99)	1.12 (0.98)	1.42 (1.36)	2.04 (1.24)	1.74 (1.22)	1.58 (0.98)
*% in group is*										
Female	61.02	71.65	58.33	67.11	55.03	45.45	63.33	60.18	60.81	61.05
Low education	48.15	62.20	38.89	53.02	47.62	16.67	61.67	60.18	52.25	30.23
Middle education	29.63	25.98	33.33	29.53	26.98	42.42	18.33	23.89	30.63	36.05
High education	22.22	11.81	27.78	17.45	25.40	40.91	20.00	15.93	17.12	33.72
Has a partner	50.62	33.86	50.00	46.31	60.32	65.15	35.00	46.90	50.90	58.14
Lives in an institution	7.58	25.20	11.11	3.36	1.06	0.00	35.00	10.62	3.15	1.74
Lung/heart/vascular disease	49.03	51.18	38.89	55.03	50.26	33.33	33.33	53.98	54.05	44.77
Diabetes	15.34	22.05	16.67	18.12	12.17	4.55	18.33	23.01	14.86	9.88
CVA	9.88	18.11	2.78	12.75	5.29	4.55	6.67	18.58	11.26	3.49
Cancer	22.75	18.11	25.00	30.87	21.69	15.15	18.33	30.09	18.92	24.42
Rheumatic disease	62.61	71.65	58.33	71.14	56.08	46.97	55.00	58.41	63.51	66.86
Deceased	24.65	56.00	22.22	18.24	17.99	0.00	61.67	41.59	17.12	10.06
Posterior probability		0.94	0.91	0.91	0.92	0.90	0.95	0.83	0.86	0.90

### Functional limitations

**Fig. 1:**
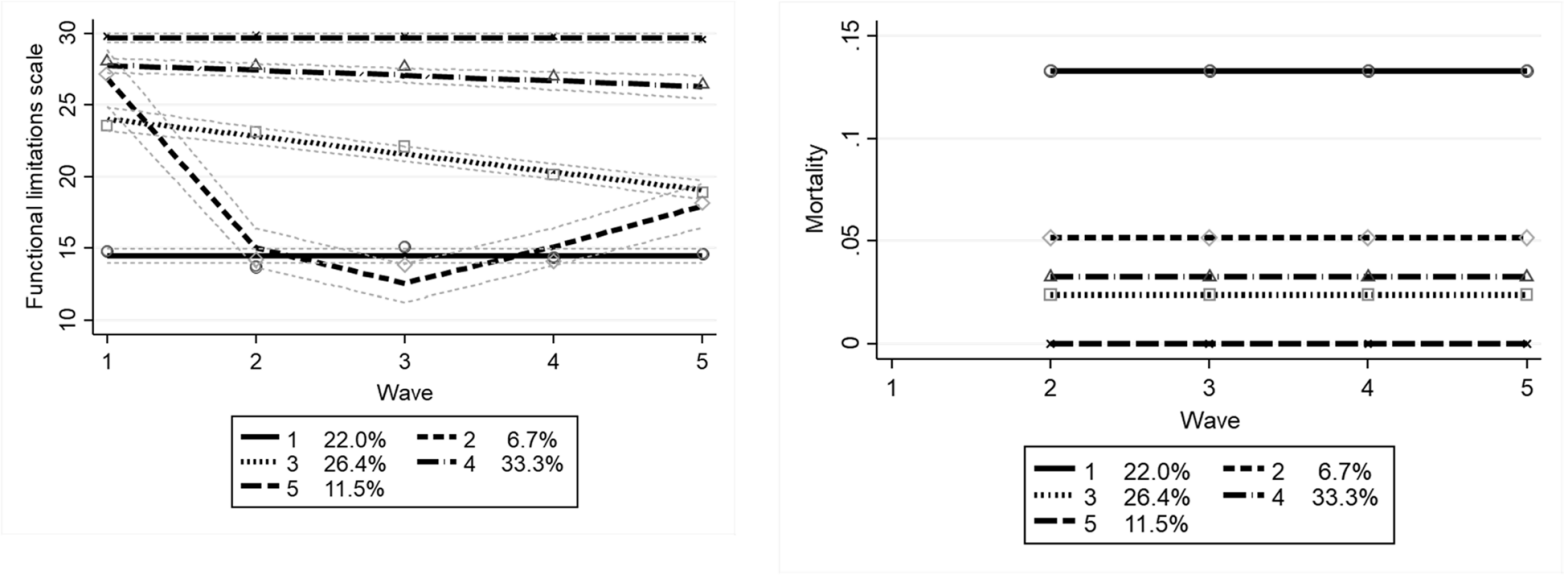
Trajectories in functional limitations (left, and mortality probability (right) by age (*N* = 567). Dotted lines show 95%-CI

**Fig. 2:**
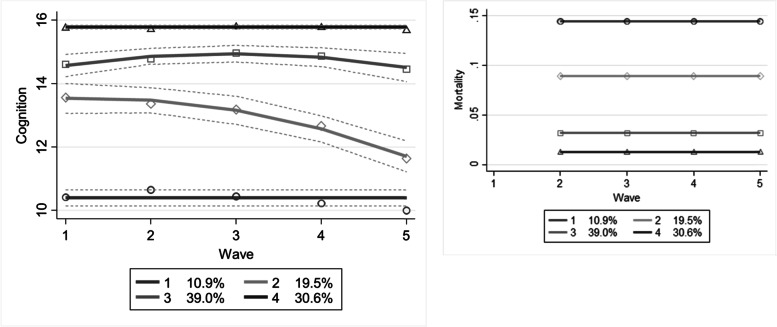
Trajectories in cognitive decline (left, and mortality probability (right) by age (*N* = 567). Dotted lines show 95%-CI

A model with five trajectories proved to be the best fit for the data (see Additional file 3 Table S1 and S2). The descriptive statistics per trajectory and the multivariate estimates are shown in Tables 3 and 4, and the trajectory plot and estimated mortality probability are shown in Fig. 1. The mortality probabilities at wave 5 in Figs. 1 and 2 should not be the same as the mortality probabilities in Table 3, since the mortality probabilities shown in Figs. 1 and 2 are probabilities of dying prior to the next survey wave based on the current survey wave.

**Table 4:** Multivariate predicted probabilities for the functional limitation trajectories (N = 567)

	*1: Continuous low functioning,* *high mortality* *(N = 127)*				*2: Steep decline in functioning, low mortality* *(N = 36)*				*3: Gradual decline,* *low mortality* *(N = 149)*				*4: Moderately high functioning, low mortality* *(N = 189)*				*5: Continuous high functioning, low mortality* *(N = 66)*			
			95% CI				95% CI				95% CI				95% CI				95% CI	
	*dy/dx (se)*	*p-value*	*LL*	*UL*	*dy/dx (se)*	*p-value*	*LL*	*UL*	*dy/dx (se)*	*p-value*	*LL*	*UL*	*dy/dx (se)*	*p-value*	*LL*	*UL*	*dy/dx (se)*	*p-value*	*LL*	*UL*
Age	**0.02 (< 0.01)**	**< 0.001**	**0.010**	**0.021**	< 0.01 (<0.01)	0.204	−0.001	0.006	**0.01 (< 0.01)**	**< 0.001**	**0.007**	**0.020**	**−0.01 (0)**	**0.001**	**−0.021**	**−0.005**	**−0.02 (< 0.01)**	**< 0.001**	**−0.025**	**−0.011**
Female	0.04 (0.04)	0.220	−0.026	0.113	−0.01 (0.02)	0.802	−0.053	0.041	0.06 (0.04)	0.131	− 0.018	0.141	− 0.05 (0.04)	0.263	− 0.134	0.037	− 0.05 (0.03)	0.081	− 0.107	0.006
Low education (ref.)																				
Middle education	−0.05 (0.04)	0.138	−0.127	0.018	0.02 (0.02)	0.382	−0.026	0.068	−0.01 (0.04)	0.886	−0.088	0.076	−0.07 (0.04)	0.129	−0.151	0.019	**0.11 (0.03)**	**< 0.001**	**0.050**	**0.162**
High education	**−0.13 (0.04)**	**0.001**	**−0.200**	**−0.050**	0.03 (0.03)	0.266	−0.024	0.089	−0.03 (0.05)	0.483	−0.126	0.060	< 0.01 (0.05)	0.929	−0.095	0.104	**0.12 (0.03)**	**< 0.001**	**0.058**	**0.186**
Has a partner	−0.04 (0.04)	0.227	−0.113	0.027	< 0.01 (0.02)	0.998	−0.046	0.046	0.02 (0.04)	0.708	−0.065	0.095	0.04 (0.04)	0.297	−0.039	0.128	−0.02 (0.03)	0.57	−0.074	0.040
Comorbidity	**0.03** **(0.01)**	**0.011**	**0.007**	**0.056**	−0.01 (0.01)	0.206	−0.030	0.007	**0.06 (0.01)**	**< 0.001**	**0.033**	**0.088**	**−0.03 (0.02)**	**0.043**	**−0.066**	**−0.001**	**− 0.05 (0.01)**	**< 0.001**	**−0.071**	**− 0.023**
Lives in an institution	**0.46 (0.08)**	**< 0.001**	**0.300**	**0.613**	0.06 (0.06)	0.291	−0.054	0.180	**−0.14 (0.06)**	**0.018**	**−0.262**	**−0.025**	**− 0.26 (0.06)**	**< 0.001**	**− 0.378**	**−0.134**	**− 0.12 (0.01)**	**< 0.001**	**−0.144**	**− 0.096**

Notes: *LL* Lower Limit, *UL* Upper Limit

The first group, containing 22.0% of the respondents, showed stable low levels of physical functioning: The trajectory started at a mean ADL-score of 15, and stayed at that level for the following waves. Such scores usually indicate that respondents were unable to perform 3 ADL-indicators, but were still able to perform 2 without help, although for some it meant that they had much difficulty performing all of the five ADL-indicators, and needed help with at least one of them. This group had the highest mortality probability per year: 13% at each wave. Older people dy/dx = 0.02 [0.01; 0.02] and people who lived in an institution dy/dx = 0.46 [0.30; 0.61] were relatively likely to follow this trajectory, as were people who suffered from multiple diseases, with each extra disease increasing the probability of this trajectory with 0.03 [0.01; 0.06]. When comparing the sample averages to the percentage of people that has a certain disease (see Table 3), the prevalence of diabetes, CVA, and rheumatic disease is markedly higher (> 5% or more) than average in this group. People with a high education were 13% [− 0.20; − 0.05] less likely compared to those with a low level of education to have a stable level of many ADL disabilities.

The second group showed a decline in functioning, followed by a slight recovery in which some of the initial functioning was regained. The decline was steep: over the course of nine months the ADL-score declined from 26 to 15, which is indicative of gaining two severe limitations. At the end of the observation period, the average ADL-score was 17. This decline might reflect the slow regaining of functioning after function loss caused by falling or an accident. The recovery is however quite small and we did not check whether participants in this trajectory experienced falls or accidents. Relatively few participants followed this trajectory (6.7%), and the mortality probability was stable at 5%. None of the included covariates were significantly associated with this trajectory, but this might be due to the small number of participants in this group. Compared to the whole sample this group had a lower percentage of CVA and lung- and heart disease.

The third trajectory showed slight decline, and gradually decreased from an average ADL-score of 24 to 19. This trajectory contained 26.4% of the participants, and had a stable mortality probability of 3%. Older people were more likely to follow this trajectory, with 1% [0.01; 0.02] extra for each life year. Participants who suffered from more diseases were 6% [0.03; 0.09] more likely to follow this trajectory, this trajectory had a higher prevalence of lung- and heart disease, cancer, and rheumatic disease. Living in an institution decreased the probability of this trajectory with dy/dx = 0.014 [− 0.26; − 0.03].

The fourth trajectory was stable, with an average ADL-score of 27, which indicated being able to perform all ADL-indicators with only a little or no help. The mortality probability was stable at 3, and 33.3% of the participants followed this trajectory. Age decreased the probability of following this trajectory with 1% [− 0.02; − 0.01] per year, as did having more diseases: dy/dx = − 0.03 [− 0.07; − 0.00].

Living in an institution was statistically significantly associated with this trajectory as well dy/dx = − 0.26 [− 0.38; − 0.13].

The fifth trajectory was stable as well, and the participants (11.5%) in this trajectory experienced no ADL-limitations at all. Older people were less likely to follow this trajectory (dy/dx = − 0.02 [− 0.03; − 0.01], as were people that suffered from more diseases, with an decrease in the probability of following this trajectory of 5% [− 0.07; − 0.02]. The prevalence of all diseases was markedly lower than average in this trajectory. Living in an institution decreased the probability of this trajectory as well, with 12% [− 0.14; − 0.10]. Education seemed to increase the probability of this favourable trajectory: the probability of this trajectory was respectively 0.11 [0.05; 0.16] and 0.12 [0.06; 0.19] higher for people with a middle education and a higher education, compared to people with a lower education.

Sensitivity analyses showed that not jointly modelling mortality resulted in different group sizes: 14% in the low (F1), 6.5% in the rapid declining (F2), 28.2% in the gradually declining (F3), 35.8% in the moderately high (F4), and 15.5% in the high (F5) trajectory (see S4 Additional file 2). This resulted in overestimations of 4, 2.5, and 1.8% of respectively the highest (F5), moderate (F4), and gradual decline (F3) physical functioning trajectories, and a − 0.2 and 8% underestimation of the rapid decline (F2) and the low physical functioning trajectory (F1), compared to the solutions of the models that included mortality.

### Cognitive limitations

For cognitive limitations a four-trajectory model, shown together with the mortality plot in Fig. 2, proved to be the best fit. The descriptive statistics per trajectory and the multivariate estimates are shown in Tables 3 and 5.

**Table 5:** Multivariate predicted probabilities for the cognitive functioning trajectories (N = 567)

	*1: Continuous low functioning. High mortality (N = 60)*				*2: Declining functioning, moderate mortality (N = 113)*				*3: Moderately functioning, low mortality (N = 222)*				*4: Continuous high functioning. Low mortality (N = 172)*			
			95% CI				95% CI				95% CI				95% CI	
	*dy/dx (se)*	*p-value*	*LL*	*UL*	*dy/dx (se)*	*p-value*	*LL*	*UL*	*dy/dx (se)*	*p-value*	*LL*	*UL*	*dy/dx (se)*	*p-value*	*LL*	*UL*
Age	< 0.01 (<0.01)	0.063	0.000	0.008	**0.01 (< 0.01)**	**< 0.001**	**0.005**	**0.017**	−0.01 (< 0.01)	0.113	−0.015	0.002	**− 0.01 (< 0.01)**	**0.027**	**− 0.016**	**− 0.001**
Female	− 0.03 (0.03)	0.359	− 0.083	0.030	− 0.02 (0.04)	0.558	− 0.097	0.052	− 0.03 (0.05)	0.552	− 0.118	0.063	0.08 (0.04)	0.061	−0.003	0.156
Low education (ref.)																
Middle education	**−0.06 (0.03)**	**0.021**	**−0.114**	**−0.009**	− 0.07 (0.04)	0.057	− 0.147	0.002	− 0.03 (0.05)	0.557	− 0.122	0.066	**0.16 (0.04)**	**< 0.001**	**0.077**	**0.247**
High education	−0.04 (0.03)	0.204	−0.099	0.021	**−0.09 (0.04)**	**0.04**	**−0.167**	**−0.004**	**− 0.14 (0.05)**	**0.007**	**− 0.237**	**−0.037**	**0.26 (0.05)**	**< 0.001**	**0.162**	**0.361**
Has a partner	−0.04 (0.03)	0.104	−0.098	0.009	0.01 (0.04)	0.734	−0.062	0.087	−0.03 (0.05)	0.525	−0.120	0.061	0.06 (0.04)	0.148	−0.022	0.144
Comorbidity	**−0.03 (0.01)**	**0.01**	**−0.049**	**−0.006**	**0.04 (0.01)**	**0.004**	**0.012**	**0.063**	0.01 (0.02)	0.642	−0.026	0.041	−0.02 (0.02)	0.256	−0.049	0.013
Lives in an institution	**0.34 (0.08)**	**< 0.001**	**0.187**	**0.497**	0.05 (0.07)	0.449	−0.083	0.187	**−0.19 (0.07)**	**0.011**	**−0.337**	**− 0.045**	**−0.20 (0.06)**	**0.001**	**−0.326**	**− 0.080**

Notes: *LL* Lower Limit, *UL* Upper Limit

The first group showed very low cognitive functioning across time, the trajectory started at the threshold for dementia [11] with a mean sMMSE of 11. The trajectory showed no further decline, but it could be argued that given the low baseline scores, there was little further decline possible for this group. Containing 10.9% of the sample, this group was the smallest of the four trajectories. The mortality probability was continuously high at 14%. Age, although showing a very slight positive direction, was not significantly statistically associated with this trajectory dy/dx < 0.01 [0.00; 0.01], which might be explained by the low power of this study, or by the fact that diseases such as Alzheimer’s usually start between age 65–75. It appears that low educated people were most likely to follow this trajectory, and this difference was significant between low and middle educated people, with middle educated people being 6% [− 0.11; − 0.01] less likely to have continuous low cognitive functioning. Suffering from more diseases decreased the probability of this trajectory with 3% [− 0.05; − 0.01], and the people in this trajectory on average had less diseases: the prevalence of lung- and heart disease, diabetes, CVA, and rheumatic disease was lower (> 5%) than the sample average. Living in an institution increased the probability with 34% [0.19; 0.50].

The second trajectory (19.5%) showed decline, decreasing from probable mild cognitive impairment (sMMSE = 13) to probable dementia (sMMSE = 11). The mortality probability was quite high: 9%. Being older increased the probability of this trajectory dy/dx = 0.01 [0.01; 0.02], as did having more diseases dy/dx = 0.04 [0.01; 0.06]. On average, this group had a higher prevalence of diabetes, CVA, and cancer. Having had a high education decreased the probability of this trajectory with 9% [− 0.17; <− 0.01].

The third group, containing 39% of the participants, started at a mean sMMSE of 14, slightly increased to 15, and then decreased to 14. It had a stable mortality probability of 4%. People with a high education, or people that lived in an institution were respectively 14% [− 0.24; − 0.04] and 19% [− 0.34; − 0.05] significantly less likely to follow this trajectory. The prevalence of lung- and heart disease was 5% higher than the sample average.

The last trajectory showed continuous high cognitive functioning combined with a low mortality probability (2%), and contained 30.6% of the participants. Older age decreased the probability of this trajectory dy/dx = − 0.01 [− 0.02; < − 0.01], as did living in an institution dy/dx = − 0.2 [− 0.33; − 0.08]. People with a middle or high education respectively had a 16% [0.08; 0.25] and 26% [0.16; 0.36]. Although the association for morbidity was not significant, the prevalence of diabetes and CVA was > 5% higher than the sample average.

Not jointly modelling mortality resulted in the following group sizes: 5.4% in the low (C1), 15.4% in the declining (C2), 43.8% in the moderate (C3), and 35.5 in the high (C4) trajectory. The high (C4) and moderate (C3) cognitive functioning trajectories would have been overestimated with 4.9 and 4.8%, while the declining (C2) and low (C1) trajectories would have been underestimated with 4.1 and 5.5%.

### Overlap between the trajectories

**Fig. 3:**
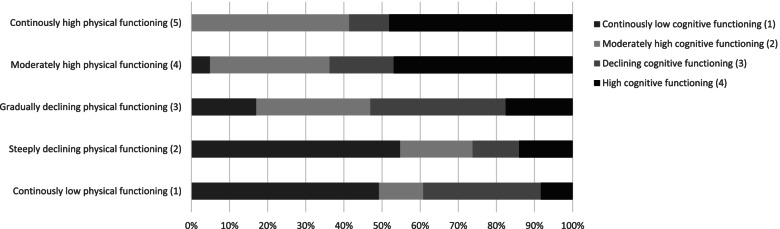
Overlap between functional limitations trajectories and cognitive trajectories: percentage of people in cognitive functioning trajectory per functional limitations trajectory

**Fig. 4:**
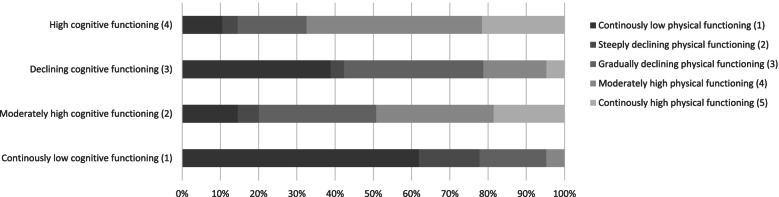
Overlap between functional limitations trajectories and cognitive trajectories: percentage of people in functional limitations trajectory per cognitive functioning trajectory

Figures 3 and 4 show overlap between the trajectories of functioning. A certain coherence is visible: people in the adverse physical functioning trajectories experience low cognitive functioning more often, and people experiencing high physical functioning often experience high cognitive functioning. However, there is also still substantial variation, since 22% of people with high cognitive function experience a trajectory with severe ADL-limitations. The picture for the declining physical trajectory shows little correlation with cognition: the percentages of the cognitive trajectories are distributed almost evenly over this group.

## Discussion

This study identified trajectories in both physical and cognitive functioning among Dutch older adults aged 75 and older. Using the innovative methodology of Group-Based Trajectory Modelling, modelling trajectories jointly with mortality, we were able to estimate more precise group sizes of the trajectories. We identified five trajectories in functional limitations and four trajectories in cognitive decline. A considerable proportion of the Dutch 75+ experienced high levels of functioning over the course of three years. For physical functioning, 11.5% of the sample experienced continuous high levels of physical functioning, and 33.3% of the sample experienced high moderate physical functioning. As for cognitive functioning, 30.6% of the sample experienced high cognitive functioning and 39% experienced moderately high cognitive functioning. But, adverse trajectories were present as well. For the physical functioning trajectories, 26.4% of the participants experienced gradual decline and 6.7% experienced steep decline followed by slight recovery. For cognitive functioning 19.5% experienced rapid cognitive decline. The most adverse trajectories showed continuous low physical functioning with at least 2 severe ADL-limitations (22%), and continuous low cognitive functioning (10.9%) with probable dementia. These trajectories had high mortality levels (~ 14%). The declining and low functioning trajectories are the trajectories where the requirement for care is probably highest. Despite our study using shorter time intervals, examining older participants, and incorporating mortality risk, the trajectories seem to reflect patterns that were also identified in previous studies on trajectories of functioning in old age. Among populations of the same age group similar trajectories were identified [14, 16]. Whereas our relatively old study sample resulted in a low trajectory for cognition, that is not identified among younger study samples [7, 8], but is also identified among older study samples [17, 46]. It can be concluded that our study corroborates that there is considerable diversity in health trajectories among the 75-plus. Taking mortality into account resulted in bigger group sizes for the more adverse trajectories, while it led to smaller group sizes for the more favourable trajectories, which is in line with what could be expected based on the studies conducted by Haviland et al. [26] and Zimmer et al. [29], who used the same methodology. However, although we expected that modelling mortality would result in bigger group sizes for the trajectories that showed decline, this was only the case for cognitive decline, but not modelling mortality only resulted in a negligibly small (0.2%) underestimation of the steeply declining physical functioning trajectory and a slight overestimation of the gradually declining physical functioning. This is in line with previous studies reporting very low mortality probabilities for people with increasing functional limitations [10].

The second aim of our study was to explore how the trajectories varied for several background variables (sex, age, level of education and partner status) and disease status. What is clear from these results is that the persons following the three most favourable trajectories (with either high or high moderate levels of physical functioning, or high levels of cognitive functioning) had rather favourable characteristics. They were younger, middle or high educated, lived independently, and had less diseases on average.

Yet, there appeared to be no common denominator between the people following the three declining trajectories. Both the gradually declining physical functioning trajectory and the gradually declining cognition trajectory shared some characteristics: higher age and a higher number of diseases increased the probability of these trajectories, they also had a higher prevalence of cancer than the sample average (> 5%), which is understandable as these chronic diseases in more advanced stages limit mobility.

The gradually declining functional limitations trajectory had more people who were living in an institution, and the gradually declining cognition trajectory had a higher diabetes and CVA prevalence. Although none of the associations for the gradually declining physical functioning trajectory were significant, these people appeared to have more favourable characteristics than the sample average: they were higher educated, had less diseases: specifically lower rates of lung- and heart disease, and CVA. This shows the severe debilitating effects of CVA, since it reduces the level of functioning in such a severe way that the chance of following a trajectory that starts with high functioning is rather low [10]. The finding that the prevalence of CVA is highest with in the gradually declining cognitive functioning is in line with previous findings that CVA does not necessarily lead to dementia, but reduces cognitive functioning, thereby resulting in mild cognitive impairment for most [47, 48]. Since CVA was measured at baseline, this is in line with the starting point of this trajectory, which indicates mild cognitive impairment.

While the prevalence of rheumatic diseases was high among the gradually declining physical functioning trajectory and low for the high physical functioning trajectory, a finding also reported by for example Botes et al. [49], the prevalence was also lower for the low cognitive functioning trajectory. This relation between rheumatic diseases and cognition has been widely studied, and despite the growing body of evidence suggesting that aspirin does not have a protective effect on cognition [50, 51], studies do indicate that non-steroidal anti-inflammatory drugs (NSAIDs) decrease the risk of cognitive decline [52].

As expected based on previous studies, older age, lower education, and living in an institution, were significantly associated with the two trajectories of poor functioning: with severe functional limitations, and with severe cognitive problems [49, 53, 54].Although both trajectories had much lower prevalence of a partner, these associations were not significant. The pattern for diseases was different from what we expected, for functional limitations more diseases was positively associated, with higher prevalence of diabetes, CVA, and rheumatic disease, which was as expected. The link with diabetes can be explained by the adverse effects of hyperglycaemia, inflammatory cytokines, and neuropathic processes [55]. For cognitive functioning the more diseases was negatively associated with the low trajectory, with lower prevalence of lung- and heart disease, and rheumatic disease. This might be because lung- and heart disease result in death before continued cognitive functioning can occur.

All in all, these trajectories seem to contain persons that experienced the deleterious effects of chronic diseases, and about half of them had to be taken into residential care due to the resulting limitations.

Associations for sex were not present. Because male brains atrophy quicker than female brains do [56], most studies stratify by sex a priori [7, 29]. However, stratifying by sex would have greatly reduced our statistical power substantially due to our small sample. And, sensitivity analysis stratified by sex showed comparable trajectories for men and women (see Figs. S2 and S3 in Additional file 2). The absence of sex differences might be explained by the finding that these differences are most pronounced in the level of functional impairment, while rates of change are similar for men and women [57]. It could be possible that due to our shorter measurement intervals the rate of change has had a bigger impact in defining the trajectories than differences in the intercept. On the other hand, the absence of sex differences is not entirely anomalous; for functional limitations Bolano et al. [24] and Holstein et al. [58] do not report any statistically significant sex differences, and Comijs et al. [8] do not always identify sex differences for trajectories in cognition. In addition, a study on the differences in cognitive decline between men and women did not find these differences [59]. Moreover, our analyses included mortality, number of chronic diseases, age and level of education, which are all factors that differ by sex, which may have decreased the effect of sex itself. Other studies focusing on the role of education have found that education may be important for onset of functional and activity limitations but not for progression [10, 60, 61]. Low education being associated with low levels of physical functioning is a finding also reported by Boyd et al. [62] and Kingston et al. [16]. The finding that a higher education is negatively associated with moderate or declining levels of cognitive functioning, and positively associated with high cognitive functioning, corroborates the link between education and cognition. Furthermore, it is partly in line with the MMSE being less sensitive for cognitive decline among higher educated people [63], but also in line with education having a protective effect on cognitive decline [64], and people with higher cognitive ability having pursued more education.

Associations for partner status were absent. This might have been caused by not differentiating between coresiding and noncoresiding partners. Second, it is possible that the protective effect of having a partner diminishes with age, since this usually results in the partner requiring more care as well. Lastly, studying a population that could be either institutionalized or community-dwelling might have resulted in absent associations for partner status.

### Strengths and limitations

The main strength of this study was the use of the 75PLUS LASA-data, containing a representative sample of the Dutch oldest old: the study has a high response and cooperation rate, and enabled for studying both community dwelling and institutionalized people. Accounting for attrition by jointly modelling mortality is a strength as well, enabling us to estimate more precise group sizes. Third, defining ADL as a scale forms a strength in opposition to previous studies that compressed the range of the severity of ADL-limitations by dichotomizing ADL. Because the overall degree of functional limitations decides the need for care, it is precisely this degree that is of vital importance for policymakers, and by measuring ADL as a scale we were better at capturing the existence and the range of need for care that follow from functional limitations. The first limitation of the study was not being able to conduct a multi-trajectory model to study the interconnectedness between cognitive decline and ADL-limitations that is implied by previous studies [65, 66]. We instead decided to report the estimates of the trajectories separately, since jointly modelling mortality in a multi-trajectory model was not possible, and accounting for decease is necessary in a very old population. Second, although the use of proxy data allowed us to also include severely cognitively impaired respondents, this resulted in two different measurements for cognition (the sMMSE and the IQCODE) [35]. Although different ways of harmonizing did not affect the trajectories much, the absence of guidelines on how to harmonize the sMMSE and IQCODE leaves some uncertainty on whether the eventual scores are an accurate reflection of cognitive functioning among our participants. Although we did not have a considerable amount of missing items for ADL or sMMSE, we are mindful of the slight overestimation of both cognitive and ADL-levels in which the imputation of these items might have resulted. On the flip side, not including these participants in the analysis would have likely resulted in an overestimation of favourable trajectories as well. Third, although our sample size was sufficient for performing analyses, analysing a bigger sample size would have allowed for performing all of the analysis stratified by sex, and would have increased statistical power. Because the dropout-function we used was pre-specified in Proc Traj, this might be a limitation. Future research could use mixed effect models with a well-specified jointly modelled missingness mechanism [67], to produce unbiased estimates, and thereby also investigate further whether the prespecified dropout function from Proc Traj produced unbiased estimates. In addition, excluding the 34 participants that dropped out due to other reasons than decease might have resulted in a selection bias, since these participants were probably less likely to experience health problems than the ones that deceased.

This study shows that age is associated with the trajectories. However, since our study design did not allow for stratifying by birth cohort, future research should focus on whether the trajectories are similar among different birth cohorts.

### Implications

This study has implications for policymakers in health and long term care. Despite this study showing that a considerably large group experiences little to no functional limitations and/ or cognitive decline, this study also identifies groups that, based on their low or declining levels of physical and cognitive functioning, have a high or increasing care need. The trajectories corresponding to the highest requirements of care are the two stable low trajectories (11–22%), and part of those groups are already living in residential care. This simultaneously shows how half of these people apparently have a high requirement of care, but do still live in independent housing, probably with a large demand on care from informal and formal caregivers. The declining trajectories (7, 20, 26%) are of most interest due to the increasing care need over time. This increase makes this group vital for policies aimed at future care planning, since they require more adjustments in care provision than the stable trajectories do. Our study does not provide one indicator to target all these groups, but shows old age, low education, a higher number of chronic diseases, as the best indicators for targeting risk groups, with cancer and rheumatic disease for functional limitations, and CVA and diabetes for cognitive decline. Future studies and policymakers should aim at finding indicators to identify the people that experience declines in functioning, and in particular rapid declines in functioning.

## Conclusions

Our study underscores the diversity in health trajectories among the older old. Most Dutch 75-plus had high levels of functioning. Yet, about a quarter of the respondents experienced moderate functional decline, while 31 and 20% experienced functional and cognitive decline. A small part experienced very low levels of functioning: 22 and 11% experienced severe functional limitations or cognitive limitations with probable dementia and high mortality probabilities. The findings show that chronic disease prevalence is different for physical and cognitive functioning, with only cancer having a high prevalence among both the functional and the cognitive decline trajectories. Older age, low education, a higher number of chronic disease. Because cancer is the most likely predictor for decline, this should be the predictor policymakers use for future care planning and identifying people at risk for adverse functioning. A small percentage of the Dutch oldest old lives independently while having a high care requirement, and a considerable number of people has an increasing care need. It is important to identify whether the groups with currently high and an increasing care requirement get the care they need.

## Supplementary Information

**Additional file 1: Figure S1**. Trajectories in cognitive decline based on two different ways of harmonizing the IQCODE and the MMSE.

**Additional file 2: Figure S2**. Trajectories in functional limitations estimated stratified by gender (*N* = 567). **Figure S3**. Trajectories in cognitive decline, estimated stratified by gender (*N* = 567). **Figure S4**. Trajectories in functional limitations and cognitive decline, for survivors or deceased participants.

**Additional file 3: Table S1**. BIC Scores for different numbers of trajectories, selected model in italics. **Table S2**. Posterior probabilities for separate analysis for different numbers of trajectories, selected number of groups in italics.

**Additional file 4.**

## Data Availability

Anonymized participant-level data can be requested from LASA using a standard analysis proposal form, available through http://www.lasa-vu.nl/data/availability_data/availability_data.htm.

## Abbreviations

ADL: Activities of daily living
AME: Average marginal effects
CVA: Cerebrovascular accidents
GBA: Gemeentelijke basisadministratie
LASA: Longitudinal Aging Study Amsterdam
sMMSE: Short Mini Mental State Examination
