# Supplementary material for: Three-year trajectories in functional limitations and cognitive decline among Dutch 75+ year olds, using nine-month intervals
Source: BMC Geriatr. 2022 Feb 1;22:89. doi: 10.1186/s12877-021-02720-x (PMC8805337; doi:10.1186/s12877-021-02720-x)
Supplement: Supplementary file 1 — Additional file 1: Figure S1. Trajectories in cognitive decline based on two different ways of harmonizing the IQCODE and the MMSE. [file 12877_2021_2720_MOESM1_ESM.docx]

**Title:** Three-year trajectories in Functional Limitations and Cognitive Decline among Dutch 75+ year olds, using nine-month intervals.

**Authors:** Maura Kyra Maria Gardeniers^1^ (corresponding author), Marjolein Irene Broese van Groenou^2^, Erik Jan Meijboom^3^, Martijn Huisman^4^

**Institutional addresses:** ^1^Vrije Universiteit Amsterdam, Department of Sociology, De Boelelaan, 1081 Amsterdam, The Netherlands. ^2^Vrije Universiteit Amsterdam, Department of Sociology, De Boelelaan, 1081 Amsterdam, The Netherlands. ^3^Vrije Universiteit Amsterdam, Department of Sociology, De Boelelaan, 1081 Amsterdam, The Netherlands. ^4^Amsterdam UMC, Vrije Universiteit Amsterdam, Department of Epidemiology & Biostatistics, Amsterdam Public Health research institute, De Boelelaan, 1117 Amsterdam, Netherlands. Vrije Universiteit Amsterdam, Department of Sociology, De Boelelaan, 1081 Amsterdam, The Netherlands.

**Correspondence to:** m.k.m.gardeniers@vu.nl

**1. Harmonizing the IQCODE and the MMSE**

| Figure S1 *Trajectories in cognition based on two different ways of harmonizing the IQCODE and the MMSE* | | |
| --- | --- | --- |
|  | MMSE-scores per wave | Mortality-probability per wave |
| *Based on Chiriboga, McHugh, and Sweeney (2008)* | | |
|  | 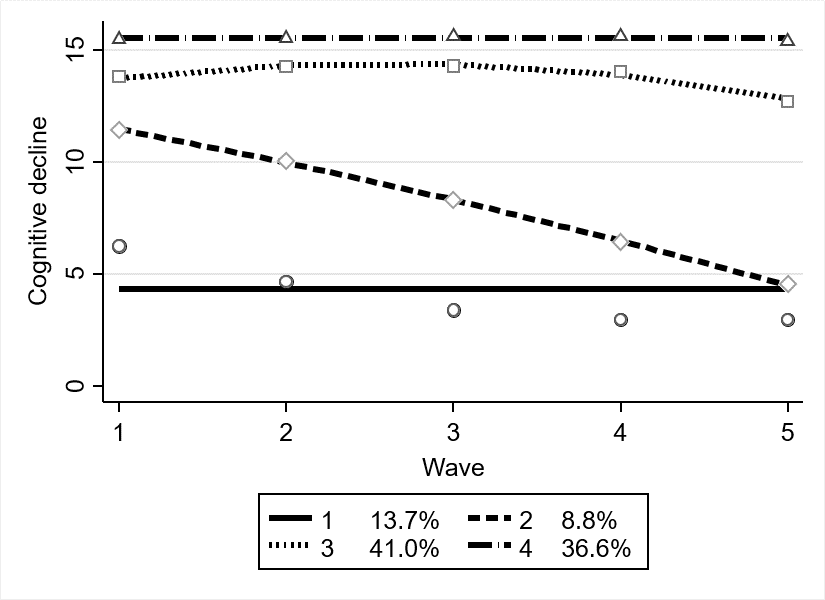 | 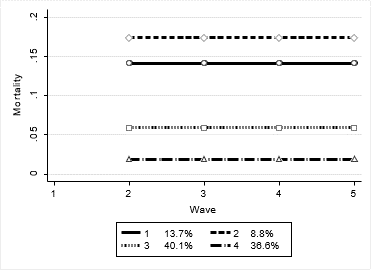 |
| *Based on van den Kommer et al. (2018)* | | |
|  | 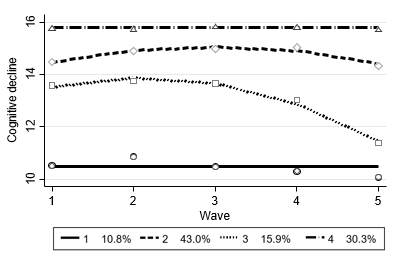 | 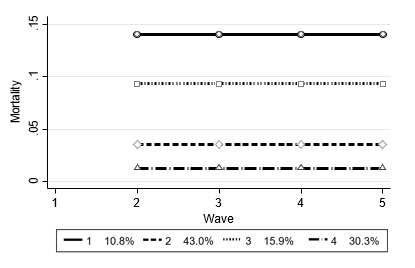 |

One of the main problems in harmonizing the IQCODE and the MMSE lies in the fact that both scales have a different time-axis; the IQCODE asks for changes in the past ten years, whereas the MMSE measures cognitive abilities in the present. However, when looking at the individual IQCODE-scores over time, there was much more variation than one might expect to be present. Since the IQCODE asks about changes in the past ten years, we would have expected that these scores would not vary substantially over the course of nine months, but for many, it did. This variation didn’t solely show decreasing cognitive abilities, but recovery as well. This seems to imply that most proxies answering the IQCODE have the respondent’s cognitive ability of the past year most vividly in mind when answering the question. With this assumption in mind, we argue that harmonizing the two is in some way justified.

There were two decisions to be made, the first one was whether we chose a conversion from the IQCODE to the MMSE at each wave, or whether we treated the IQCODE-score as a score that had to be interpreted as a change of the respondent’s previous cognitive ability score. So, in other words: whether we decided someone’s MMSE-score would be the previous MMSE-score minus a certain amount of points (based on the IQCODE, that either indicated no/ a little/ or drastic decline), or whether we calculated the MMSE-score based on the current IQCODE-score (so without the cognitive-ability-score of the previous wave in mind). Although the option of using the IQCODE to calculate the MMSE based on the previous cognitive score seemed more correct (since it asks about cognitive change), this lead to a small group of respondents ending up with extremely low or even negative scores. In addition, because an MMSE-score below 11 indicates dementia, we assume that the difference between an MMSE-core of 8 and a score of 6 is less meaningful than the difference between a score of 16 (no cognitive decline) and 14 (where mild cognitive impairment is likely to be present). Therefore we decided to harmonize the IQCODE and the MMSE without treating the IQCODE as a measure of change compared to previous cognitive statuses, but instead treating it as an indication of the respondents’ current cognitive abilities.

When looking at the trajectories that are estimated based on the two possible ways of converting the IQCODE-scores to the MMSE-scores, shown in figure 1, they do not seem to substantially differ from one another. The conversion based on the research conducted by Chiriboga, Mchugh, and Sweeney (1) is almost similar to the one based on the research conducted by van den Kommer et al. (2).

1. Chiriboga DA, Mchugh D, Sweeney MA. Clinical Gerontologist The Mini-Mental Exam (Mini-ME) An Unobtrusive and Brief Test for Cognitive Problems? 2008 [cited 2021 Jun 11]; Available from: https://doi.org/10.1300/J018v27n01_02

2. Van Den Kommer TN, Deeg DJH, Van Der Flier WM, Comijs HC. Time Trend in Persistent Cognitive Decline: Results from the Longitudinal Aging Study Amsterdam. Journals Gerontol - Ser B Psychol Sci Soc Sci. 2018;73(April):S57–64.
