# Supplementary material for: Three-year trajectories in functional limitations and cognitive decline among Dutch 75+ year olds, using nine-month intervals
Source: BMC Geriatr. 2022 Feb 1;22:89. doi: 10.1186/s12877-021-02720-x (PMC8805337; doi:10.1186/s12877-021-02720-x)
Supplement: Supplementary file 2 — Additional file 2: Figure S2. Trajectories in functional limitations estimated stratified by gender (N = 567). Figure S3. Trajectories in cognitive decline, estimated stratified by gender (N = 567). Figure S4. Trajectories in functional limitations and cognitive decline, for survivors or deceased participants. [file 12877_2021_2720_MOESM2_ESM.docx]

**Title:** Three-year trajectories in Functional Limitations and Cognitive Decline among Dutch 75+ year olds, using nine-month intervals.

**Authors:** Maura Kyra Maria Gardeniers^1^ (corresponding author), Marjolein Irene Broese van Groenou^2^, Erik Jan Meijboom^3^, Martijn Huisman^4^

**Institutional addresses:** ^1^Vrije Universiteit Amsterdam, Department of Sociology, De Boelelaan, 1081 Amsterdam, The Netherlands. ^2^Vrije Universiteit Amsterdam, Department of Sociology, De Boelelaan, 1081 Amsterdam, The Netherlands. ^3^Vrije Universiteit Amsterdam, Department of Sociology, De Boelelaan, 1081 Amsterdam, The Netherlands. ^4^Amsterdam UMC, Vrije Universiteit Amsterdam, Department of Epidemiology & Biostatistics, Amsterdam Public Health research institute, De Boelelaan, 1117 Amsterdam, Netherlands. Vrije Universiteit Amsterdam, Department of Sociology, De Boelelaan, 1081 Amsterdam, The Netherlands.

**Correspondence to:** m.k.m.gardeniers@vu.nl

**Sensitivity checks**

**1.1 stratified by gender (Figure S2 and S3)**

The first sensitivity check we conducted was estimating the models stratified by gender, these trajectories are shown in figures S2 and S3. It has to be noted that for functional limitations the second group for men is not present among the women’s trajectories. However, it is only the first wave in which these two trajectories differ substantially, since they hoover around a mean ADL-score of 15 from the second wave onwards. Because this group is small, it is also likely that a similar pattern is present for women but cannot be detected due to the small sample size. Since the other trajectories for functional limitations and all the trajectories for cognitive decline are similar for men and women, we decided that the power of performing the analyses on a non-stratified sample, outweighed the fact that there was one trajectory among men and women that was not entirely comparable.

| **Figure S2** *Trajectories in functional limitations estimated stratified by gender (N=567)* | | |
| --- | --- | --- |
|  | Men (N=221) | Women (N=346) |
| *Trajectories in functional limitations* | | |
|  | 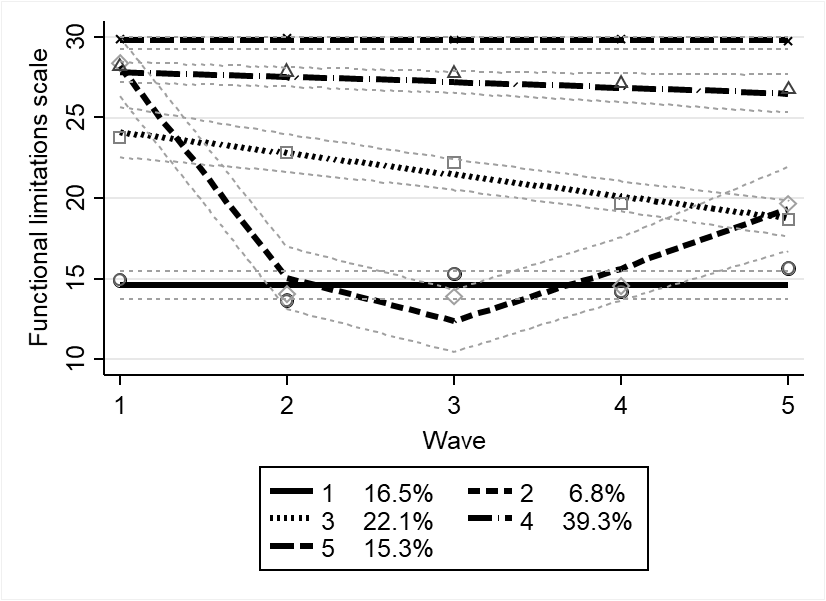 | 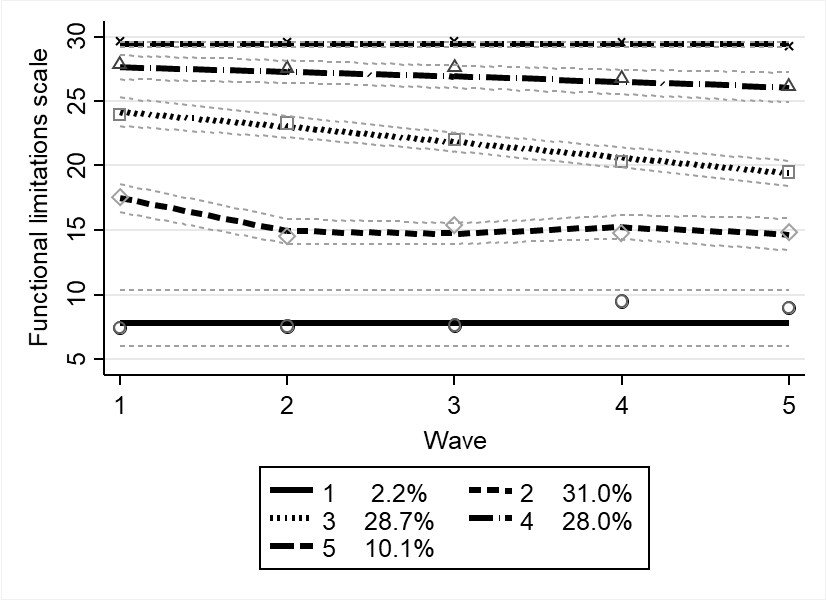 |
| *Estimated mortalityprobabilities for the functonal limitations trajectories* | | |
|  | 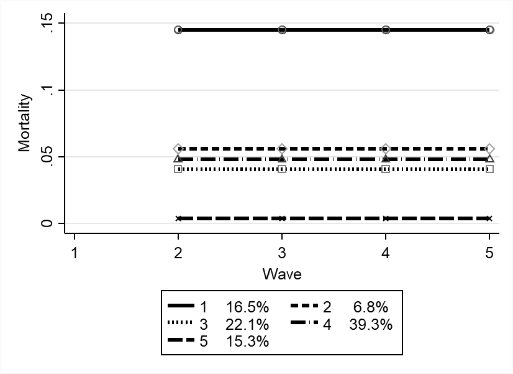 | 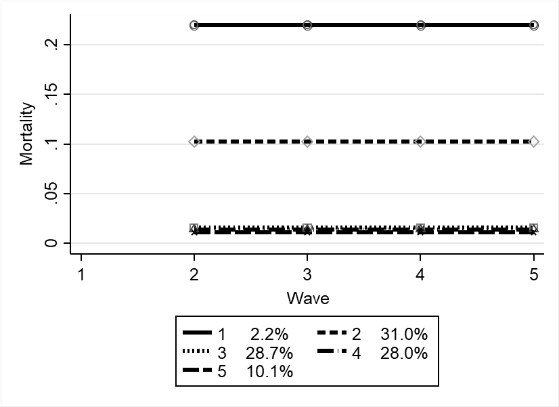 |

| **Figure S3** *Cognitive decline trajectories estimated stratified by gender (N=567)* | | |
| --- | --- | --- |
|  | Men (N=221) | Women (N=346) |
| *Trajectories in cognitive decline* | | |
|  | 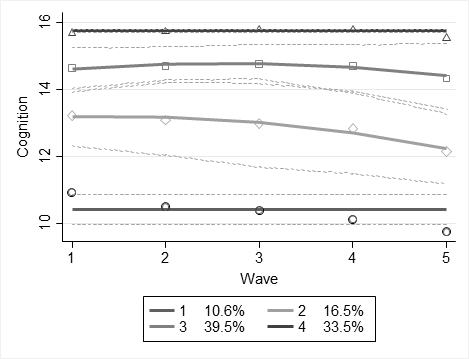 | 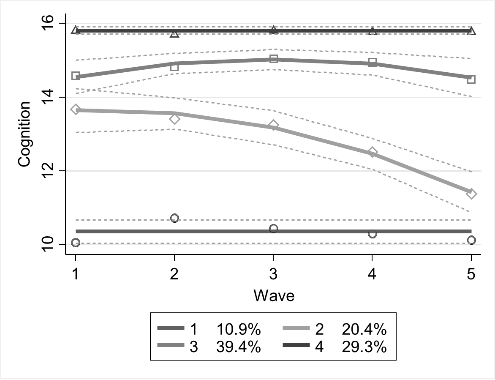 |
| *Estimated mortalityprobabilities for the cognitive decline trajectories* | | |
|  | 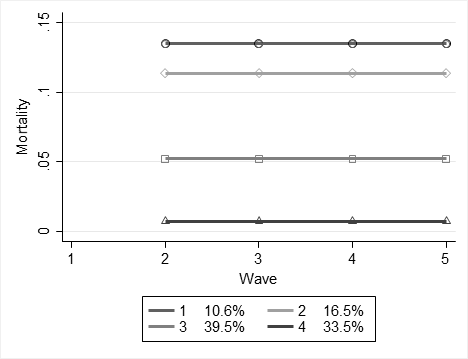 | 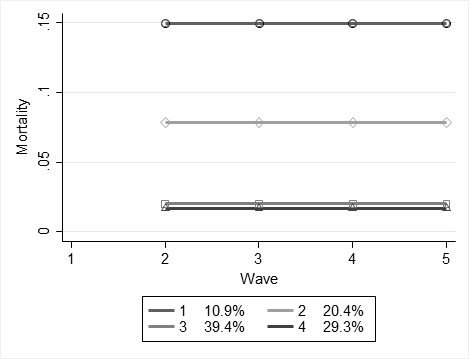 |
|  | | |

**1.2 Stratified based on mortality**

| Figure S4 *Trajectories in functional limitations and cognitive decline, for survivors or deceased participants* | | |
| --- | --- | --- |
|  | Survivors (N=425) | Deceased (N=139) |
| *Trajectories in functional limitations* | | |
|  | 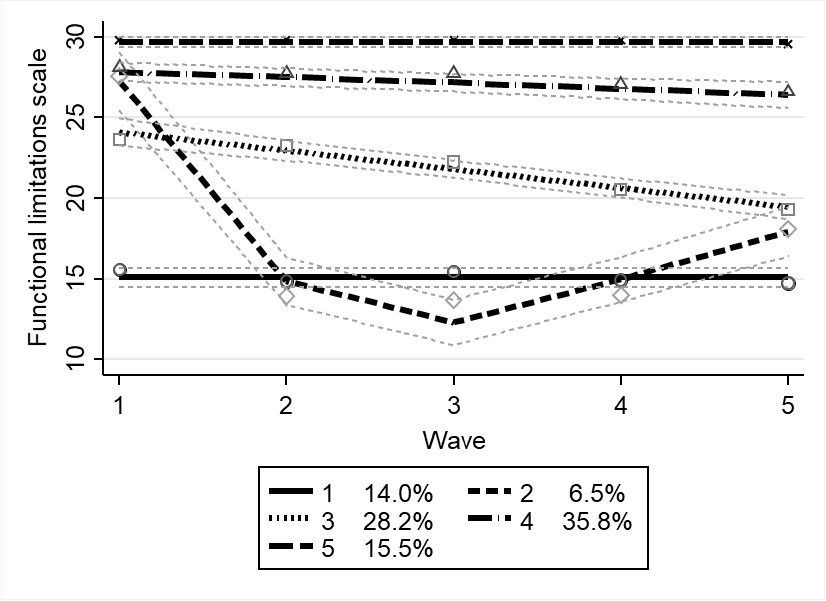 | 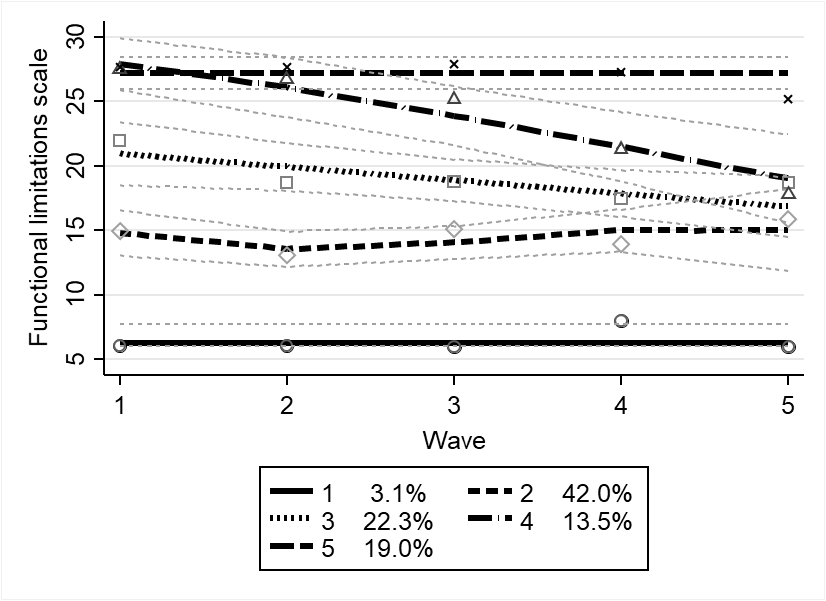 |
| *Trajectories in cognitive decline* | | |
|  | 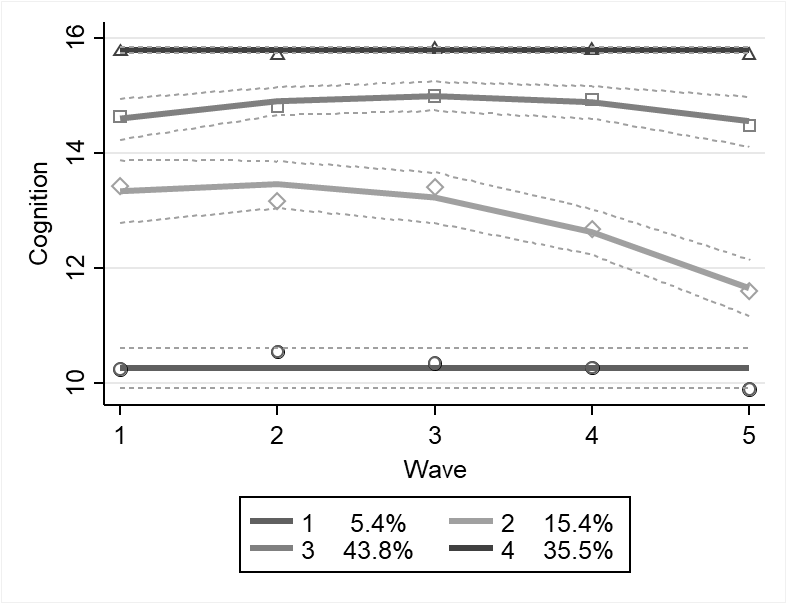 | 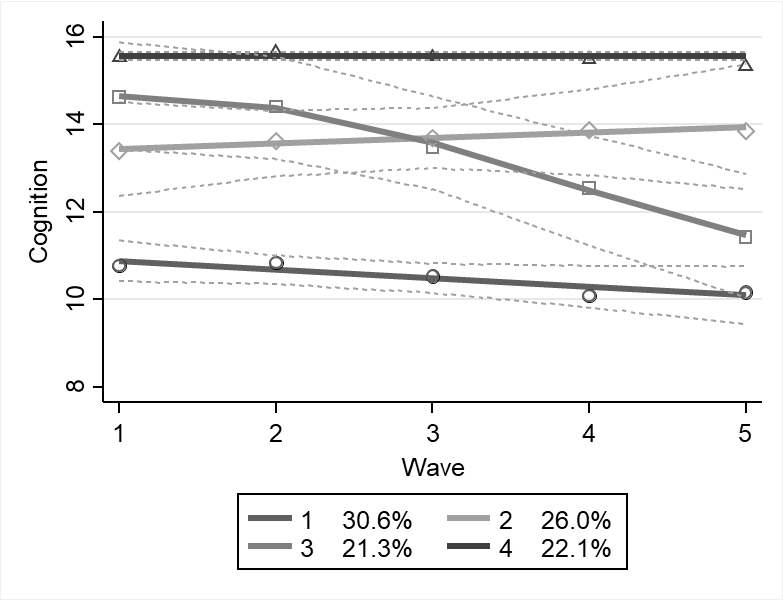 |
|  | | |
