# Supplementary material for: Three-year trajectories in functional limitations and cognitive decline among Dutch 75+ year olds, using nine-month intervals
Source: BMC Geriatr. 2022 Feb 1;22:89. doi: 10.1186/s12877-021-02720-x (PMC8805337; doi:10.1186/s12877-021-02720-x)
Supplement: Supplementary file 3 — Additional file 3: Table S1. BIC Scores for different numbers of trajectories, selected model in italics. Table S2. Posterior probabilities for separate analysis for different numbers of trajectories, selected number of groups in italics. [file 12877_2021_2720_MOESM3_ESM.docx]

**Title:** Three-year trajectories in Functional Limitations and Cognitive Decline among Dutch 75+ year olds, using nine-month intervals.

**Authors:** Maura Kyra Maria Gardeniers^1^ (corresponding author), Marjolein Irene Broese van Groenou^2^, Erik Jan Meijboom^3^, Martijn Huisman^4^

**Institutional addresses:** ^1^Vrije Universiteit Amsterdam, Department of Sociology, De Boelelaan, 1081 Amsterdam, The Netherlands. ^2^Vrije Universiteit Amsterdam, Department of Sociology, De Boelelaan, 1081 Amsterdam, The Netherlands. ^3^Vrije Universiteit Amsterdam, Department of Sociology, De Boelelaan, 1081 Amsterdam, The Netherlands. ^4^Amsterdam UMC, Vrije Universiteit Amsterdam, Department of Epidemiology & Biostatistics, Amsterdam Public Health research institute, De Boelelaan, 1117 Amsterdam, Netherlands. Vrije Universiteit Amsterdam, Department of Sociology, De Boelelaan, 1081 Amsterdam, The Netherlands.

**Correspondence to:** m.k.m.gardeniers@vu.nl

**1. Measures of for different number of groups**

| **Table S1**  BIC Scores for different numbers of trajectories, selected model in italics | | | | |
| --- | --- | --- | --- | --- |
| No. Of groups | Functional limitations | | Cognitive decline | |
|  | N=567  subjects | N= 2626 observations | N=567 subjects | N=2625 observations |
| 3 | -7569.02 | -7578.22 | -4647.63 | -4656.83 |
| 4 | -7470.78 | -7483.81 | *-4604.83* | *-4617.09* |
| 5 | *-7392.96* | *-7408.29* | -4611.16 | -4627.26 |
| 6 | -7395.52 | -7413.15 | -4619.42 | -4637.81 |

| **Table S2**  Posterior probabilities for separate analysis for different numbers of trajectories, selected number of groups in italics | | | | | | | | | | | | |
| --- | --- | --- | --- | --- | --- | --- | --- | --- | --- | --- | --- | --- |
| Functional limitations | | | | | | | | | | | | |
|  | 1 | N | 2 | N | 3 | N | 4 | N | 5 | N | 6 | N |
| 3 groups | .95 | 176 | .90 | 187 | .95 | 204 |  |  |  |  |  |  |
| 4 groups | .95 | 157 | .89 | 157 | .90 | 184 | .87 | 69 |  |  |  |  |
| *5 groups* | *.94* | *127* | *.90* | *36* | *.91* | *149* | *.92* | *188* | *.90* | *67* |  |  |
| 6 groups | .98 | 6 | .94 | 157 | .87 | 132 | .83 | 18 | .91 | 188 | .90 | 66 |
| Cognitive functioning | | | | | | | | | | | | |
|  | 1 | N | 2 | N | 3 | N | 4 | N | 5 | N | 6 | N |
| 3 groups | .95 | 80 | .89 | 242 | .94 | 245 |  |  |  |  |  |  |
| *4 groups* | *.95* | *60* | *.84* | *113* | *.86* | *222* | *.90* | *172* |  |  |  |  |
| 5 groups | .95 | 56 | .76 | 42 | .75 | 124 | .83 | 184 | .83 | 161 |  |  |
| 6 groups | .96 | 56 | .71 | 45 | .76 | 93 | .74 | 199 | .86 | 117 | .57 | 57 |
|  |  |  |  |  |  |  |  |  |  |  |  |  |
