# Supplementary material for: Three-year trajectories in functional limitations and cognitive decline among Dutch 75+ year olds, using nine-month intervals
Source: BMC Geriatr. 2022 Feb 1;22:89. doi: 10.1186/s12877-021-02720-x (PMC8805337; doi:10.1186/s12877-021-02720-x)
Supplement: Supplementary file 4 — Additional file 4. [file 12877_2021_2720_MOESM4_ESM.docx]

**Title:** Three-year trajectories in Functional Limitations and Cognitive Decline among Dutch 75+ year olds, using nine-month intervals.

**Authors:** Maura Kyra Maria Gardeniers^1^ (corresponding author), Marjolein Irene Broese van Groenou^2^, Erik Jan Meijboom^3^, Martijn Huisman^4^

**Institutional addresses:** ^1^Vrije Universiteit Amsterdam, Department of Sociology, De Boelelaan, 1081 Amsterdam, The Netherlands. ^2^Vrije Universiteit Amsterdam, Department of Sociology, De Boelelaan, 1081 Amsterdam, The Netherlands. ^3^Vrije Universiteit Amsterdam, Department of Sociology, De Boelelaan, 1081 Amsterdam, The Netherlands. ^4^Amsterdam UMC, Vrije Universiteit Amsterdam, Department of Epidemiology & Biostatistics, Amsterdam Public Health research institute, De Boelelaan, 1117 Amsterdam, Netherlands. Vrije Universiteit Amsterdam, Department of Sociology, De Boelelaan, 1081 Amsterdam, The Netherlands.

**Correspondence to:** m.k.m.gardeniers@vu.nl

We used the dropout function provided in Proc Traj, described in further detail as equation 3, in the article written by Haviland, Jones, and Nagin (2011) on page 372 (26), and as equation 4 in Zimmer et al. (2012) on page 302 (29). Dropout was described by the following probability function:

*
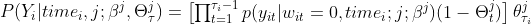
*

The distribution of the outcome was denoted by P(Y_i_|time_i_), with *Y_i_* capturing the longitudinal sequence of the participant’s ADL-score or MMSE-score*,* and time_i_ representing the wave these scores were recorded. The vector β^j^ determined the shape of the trajectory of group *J,* and θ^j^_τ_ the dropout probability of group *J* over the *τ* periods of measurement*.* The part of the equation between brackets denoted the probability of Y_i_ for each period given group J, until the moment of dropout τ_i_. This was multiplied by the probability of the dropout θ*^j^_τ_* of group J, at time τ_i_. And where *p(*)* was the distribution of *y_it_* conditional on group *j* and the wave of the data of individual *i* at time *t*, with *y_it_* being the participants ADL-score or MMSE-score, or in the case of dropout a value indicating dropout. The dropout indicator was *w_it_*, having the value 1 if the individual dropped out.
